# Analysis of Genetic Variants Associated with COVID-19 Outcome Highlights Different Distributions among Populations

**DOI:** 10.3390/jpm12111851

**Published:** 2022-11-05

**Authors:** Carlo Fabrizio, Andrea Termine, Valerio Caputo, Domenica Megalizzi, Giulia Calvino, Giulia Trastulli, Arcangela Ingrascì, Simona Ferrante, Cristina Peconi, Angelo Rossini, Antonino Salvia, Carlo Caltagirone, Claudia Strafella, Emiliano Giardina, Raffaella Cascella

**Affiliations:** 1 Data Science Unit, IRCCS Santa Lucia Foundation c/o CERC, 00143 Rome, Italy; 2 Genomic Medicine Laboratory UILDM, IRCCS Santa Lucia Foundation, 00179 Rome, Italy; 3 Medical Services Direction, IRCCS Santa Lucia Foundation, 00179 Rome, Italy; 4 Department of Clinical and Behavioral Neurology, IRCCS Santa Lucia Foundation, 00179 Rome, Italy; 5 Department of Biomedicine and Prevention, Tor Vergata University, 00133 Rome, Italy; 6 Department of Biomedical Sciences, Catholic University Our Lady of Good Counsel, 1000 Tirana, Albania

**Keywords:** COVID-19, SARS-CoV-2, populations, genetic distribution, personalized programs, Coronavirus Disease 19 outcomes

## Abstract

The clinical spectrum of SARS-CoV-2 infection ranges from asymptomatic status to mild infections, to severe disease and death. In this context, the identification of specific susceptibility factors is crucial to detect people at the higher risk of severe disease and improve the outcome of COVID-19 treatment. Several studies identified genetic variants conferring higher risk of SARS-CoV-2 infection and COVID-19 severity. The present study explored their genetic distribution among different populations (AFR, EAS, EUR and SAS). As a result, the obtained data support the existence of a genetic basis for the observed variability among populations, in terms of SARS-CoV-2 infection and disease outcomes. The comparison of ORs distribution for genetic risk of infection as well as for disease outcome shows that each population presents its own characteristics. These data suggest that each country could benefit from a population-wide risk assessment, aimed to personalize the national vaccine programs and the preventative measures as well as the allocation of resources and the access to proper therapeutic interventions. Moreover, the host genetics should be further investigated in order to realize personalized medicine protocols tailored to improve the management of patients suffering from COVID-19.

## 1. Introduction

The last COVID-19 (Coronavirus Disease 19) epidemiological update refers to the fact that 621 million individuals have been globally infected by the SARS-CoV-2 (Severe Acute Respiratory Syndrome Coronavirus-2) and the deaths accounts for 6,5 million up to 16 October 2022 [1,2]. SARS-CoV-2 is an enveloped virus characterized by a positive-sense single-stranded RNA genome of about 30 kb. The virus replicates, employing the transcription of negative-sense RNA intermediates, providing templates for the synthesis of novel genomes as well as subgenomic RNAs [3,4]. These types of RNA genomes originate from the discontinuous transcription that occurs during the synthesis of negative-strand RNA. This replicative mechanism, base editing and nucleic acid damage allow the virus to evolve speedily, by introducing genetic mutations [5]. In fact, genetic studies performed on SARS-CoV-2 genome, revealed the presence of about 29,000 Single Nucleotide Polymorphisms (SNPs) and over 10,000 insertion/deletions (indel). These data confirmed the high mutation rate and the genetic diversity of SARS-CoV-2 [6]. The World Health Organization (WHO) categorized SARS-CoV-2 genetic mutations as Variants of Concern (VOC, including Alpha, Beta, Gamma, Delta and Omicron), Variants of Interest (VOI, such as Lambda and Mu) and Variants Under Monitoring (VUM). It is important to remark that some genetic variants occurred simultaneously in the genomes of SARS-CoV-2 [6,7]. The genetic variability of SARS-CoV-2 could also have an effect on the manifestation of COVID-19 symptoms. The clinical spectrum of SARS-CoV-2 infection ranges from asymptomatic status to mild respiratory tract infections and influenza-like illness, to severe disease characterized by lung injury, multi organ dysfunction and eventually death [8,9]. The most common COVID-19 symptoms are fever, cough, fatigue, loss of taste or smell, sore throat, headache and diarrhea, while the less common signs include cutaneous rash and irritated eyes. In the last two years, the comprehension of different clinical features and the identification of specific susceptibility factors were crucial to detect people at the highest risk of severe disease and improve the outcome of COVID-19 treatment. On this subject, the main risk factors associated with severe disease are male sex, older age, ethnicity, obesity, cardiovascular and respiratory diseases and cancer [10]. Moreover, host genetic architecture has also been reported to modify the risk of SARS-CoV-2 infection and COVID-19 severity [11]. In fact, Genome Wide Association Studies (GWAS) allowed for the identification of common genetic variants conferring higher risk to SARS-CoV-2 infection and COVID-19 severity, whereas exome and genome sequencing allowed for the detection of rare genetic variants associated with both conditions. Although the genetic susceptibility to SARS-CoV-2 infection and severe COVID-19 are not completely understood, these results encourage the research for biomarkers able to predict the risk of individuals at highest risk of severe disease and improve the management and treatment of patients suffering from COVID19. On this subject, a recent work performed a GWAS meta-analysis with the purpose of identifying additional genetic variants associated with COVID-19 and testing the possible application of Genetic Risk Score (GRS) to detect high-risk patients, who may benefit from vaccination and therapeutic approaches in order to prevent severe disease and harmful complications. This study identified a rare variant (rs190509934) near the *ACE2* gene able to reduce the risk of SARS-CoV-2 infection by 40% and replicated the association of six common variants (rs73064425, rs2531743, rs143334143, rs879055593, rs2109069 and rs2236757) with susceptibility to infection, which are located in/near *LZTFL1*, *SLC6A20*, *MHC*, *ABO*, *DPP9* and *IFNAR2*, respectively [12]. In addition, four of them (rs73064425, rs143334143, rs2109069 and rs2236757) were also shown to modulate disease severity among cases. Moreover, a number of studies showed a different response to Sars-Cov-2 infection and clinical outcome across countries [13,14,15]. In this context, the different distribution of the associated genetic variants among different populations could contribute to determinine the risk of infection and disease outcomes. Given these premises, the present study aims to explore the genetic distribution of the previously mentioned variants (rs73064425, rs2531743, rs143334143, rs879055593, rs2109069 and rs2236757) among different populations and to compare their corresponding risk estimates related to the susceptibility to infection and severe COVID19 outcomes. In particular, non-parametric statistical testing was designed in order to highlight the existence of genetic variability among populations, associated with different COVID-19 outcomes. 

## 2. Materials and Methods

We downloaded Ensembl genotype data for the African (AFR), East Asian (EAS), European (EUR) and South Asian (SAS) populations using the Ensembl REST API with the GET method in Python (accessed on: 25/07/2022) [16]. This API allows the user to access Ensembl data using any programming language. The data included genotype information for each SNP, reported in Horowitz et al., [12]. The selected SNPs are: rs143334143, rs2109069, rs2236757, rs2531743, rs73064425 and rs879055593. This set of SNPs has been retrieved by the recent GWAS meta-analysis, which confirmed their association with COVID19 susceptibility and severity [12].

COVID-19 outcomes have been classified in three categories, namely: COVID-19_positive vs. COVID-19_negative (infection risk), COVID-19 severe symptoms vs. COVID-19_negative (severe illness) and COVID-19 hospitalized vs. COVID-19_negative (COVID-19 hospitalized). These categories refer to the data reported in Horowitz et al. [12].

We used the ORs generated by Horowitz et al., 2022 to evaluate if the collected populations are characterized by a differential susceptibility to COVID-19 and if the SNPs were able to identify populations with different symptoms severity. We retrieved the OR data from the Supplementary Table S12 in Horowitz et al., 2022 [12], and we computed the reciprocal OR. Having now an OR for both the effect and the reference alleles, we calculated a combined OR for each subject based on the COVID-19-associated SNPs set, as follows:

$${\mathrm{OR}}_{\mathrm{combined}}=\overset{n}{\underset{k=1}{\prod }}{\mathrm{OR}}_{k}$$

where *n* is the number of elements in the SNPs set and *k* is the elements index.

In particular, the equation explains how the combined OR was calculated for each subject. In fact, different subjects can have different combinations of OR values for each of the considered phenotypes (infection risk, severe illness, COVID-19 hospitalized), because each subject carries the reference or the effect allele. Thus, for each phenotype, the product of all ORs can have n^m^ possible values, where n is the number of possible OR values for a SNP for a phenotype (effect allele/OR, reference allele/reciprocal OR) and m is the number of SNPs considered. Hence, there are 2^6^ = 64 possible combined OR values for each phenotype. For further details and a step-by-step example calculation of the combined OR see Supplementary Materials.

The combined OR indicates the overall risk of the outcome. Between-groups comparisons in a hypothesis-testing framework on this variable assess differences in outcome susceptibility.

### Statistical Analysis

We compared the combined OR across each disease phenotype and between populations in a 3 × 4 factorial design (3 phenotypes: infection risk, COVID-19 hospitalized and severe illness; 4 populations: AFR, EAS, EUR, SAS). We tested if different populations have a different susceptibility to COVID-19 risk phenotypes as well as if the combined OR was able to differentiate populations in the symptoms severity. We used multiple two-tailed Wilcoxon tests with false discovery rate correction of *p*-value (*p*) for multiple testing [17], setting *α* = 0.05. Finally, we calculated the percentage of subjects with a risk genotype for each population and plotted differences in data distributions using boxplots and density plots with a Gaussian kernel. A density plot is based on a density function that computes and draws a kernel density estimate as a smoothed version of the histogram.

## 3. Results and Discussion

COVID-19 clinical manifestations include a wide range of symptoms ranging from mild features to severe illness and death. This phenotypic heterogeneity posed several challenges to the management of COVID-19 patients and the global healthcare systems. On this subject, several studies showed that the clinical variability could depend on different susceptibility factors including genetic and non-genetic features. To this purpose, the knowledge of the genetic architecture of the host in different populations could be advantageous for applying a personalized management of the disease, proposing population-specific intervention protocols. Given these premises, the present study has been developed with the aim of identifying differences among populations in terms of COVID-19 outcomes. In particular, the genetic distribution of six combined SNPs has been evaluated among African (AFR), East Asian (EAS), European (EUR) and South Asian (SAS) populations. The set of SNPs (rs73064425, rs2531743, rs143334143, rs879055593, rs2109069 and rs2236757) has been selected in relation to their possible phenotypic effect taking into account the results obtained by Horowitz et al.,2022 [12]. In fact, these SNPs are associated with different COVID-19 outcomes, which have been classified in three categories, namely: COVID-19_positive vs. COVID-19_negative (infection risk), COVID-19 severe symptoms vs. COVID-19_negative (severe illness) and COVID-19 hospitalized vs. COVID-19_negative (COVID-19 hospitalized). Given these premises, we decided to utilize these six SNPs to compare their distribution among different populations and their related-risk for COVID-19 outcomes independently according to age, socioeconomic status, exposure to the virus related to occupation and any other external factor. Although other SNPs have also been associated with COVID-19, we employed only these SNPs, because they have been replicated and validated by GWAS meta-analysis and, thus, they were more appropriate for the purpose of our work. Statistical analysis revealed significant differences concerning the distribution of associated variants among AFR, EAS, EUR and SAS populations (Table 1).

**Figure 1:**
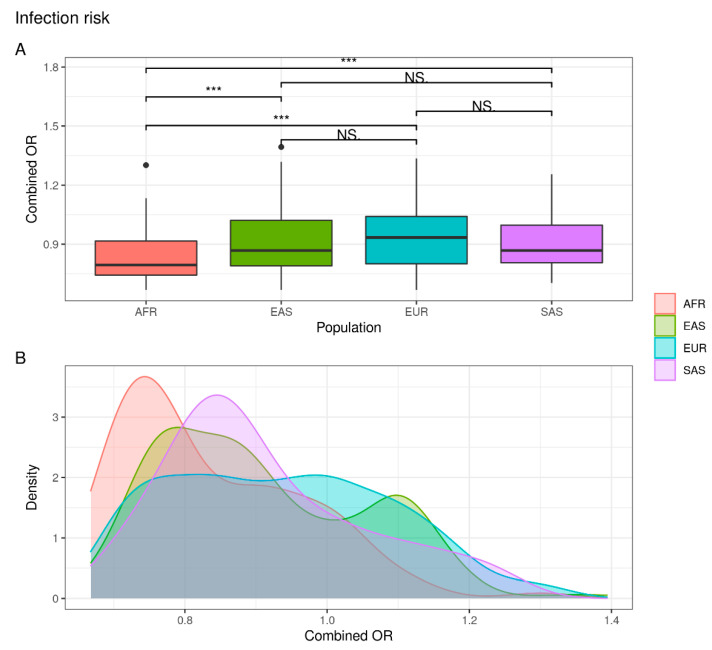
Combined OR distribution in different populations. (**A**) The boxplot reports differences between populations assessed with the Wilcoxon test (NS: not significant; *** *p* < 0.001). (**B**) The density plot highlights areas where the combined OR has a higher frequency.

The comparison of variations associated with risk of infection revealed the existence of differences in terms of genetic distribution between AFR and EAS/EUR/SAS. In fact, the frequency of individuals that overcome the OR threshold value (OR > 1) is similar between EAS, EUR and SAS populations (EAS = 32.0%; EUR = 40.3%; SAS = 29.4%) but lower in the African population (AFR = 14.6%) (Supplementary Table S1). As a result, these data suggest that the African population appears to be at lower risk for SARS-CoV-2 infection than East Asian, European and South Asian populations (Figure 1).

**Figure 2:**
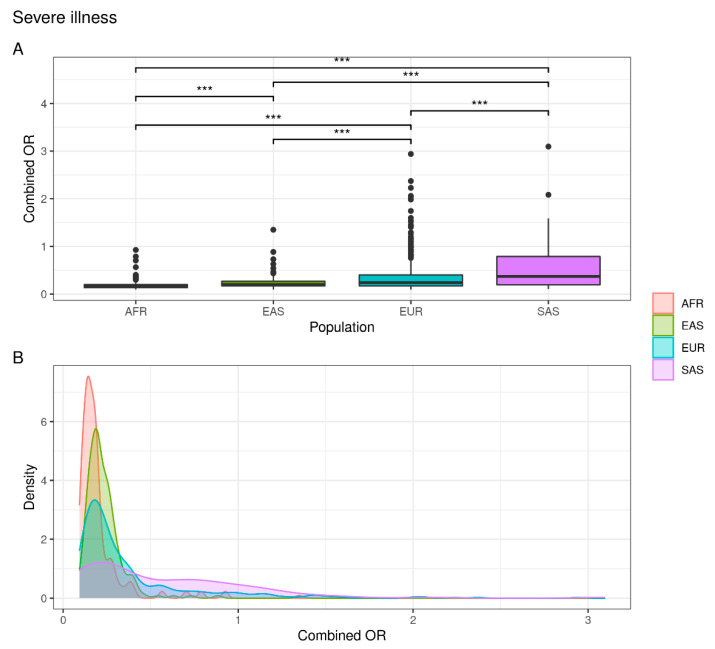
Combined OR distribution in different populations. (**A**) The boxplot reports differences between populations assessed with the Wilcoxon test (NS: not significant; *** *p* < 0.001). (**B**) The density plot highlights areas where the combined OR has a higher frequency.

As represented in Figure 2, the European (7%) and South Asian (17%) populations are at greater risk of developing severe symptoms of disease with respect to a percentage of 0.5% and 0% in Asian and African populations, respectively (Supplementary Table S1).

These data suggest that the European and South Asian populations are at greater risk of developing a severe form of the disease than the African and East Asian populations.

**Figure 3:**
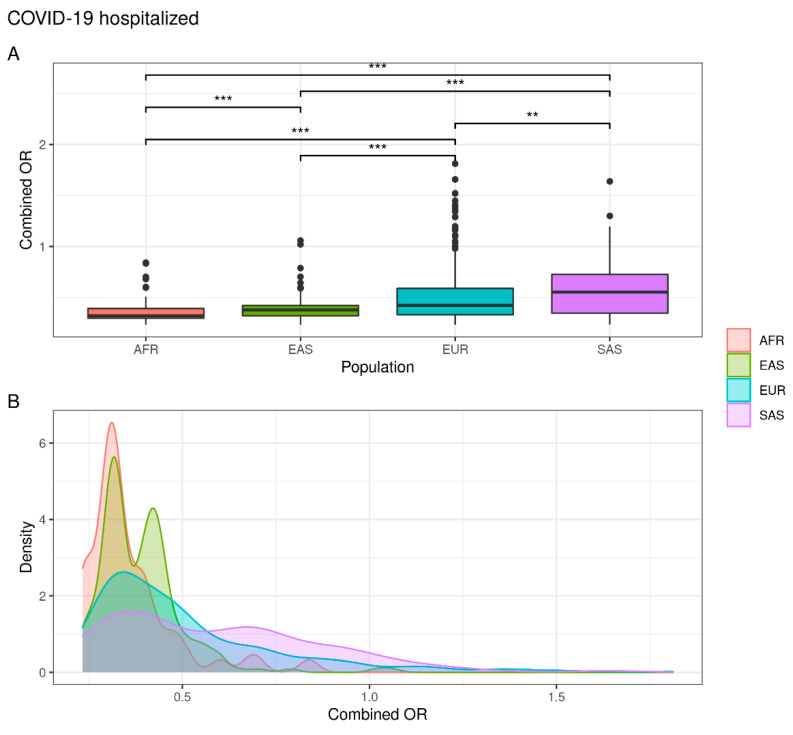
Combined OR distribution in different populations. (**A**) The boxplot reports differences between populations assessed with the Wilcoxon test (NS. not significant; ** *p* < 0.01; *** *p* < 0.001). (**B**) The density plot highlights areas where the combined OR has a higher frequency.

As expected, the genetic distribution of combined SNPs in COVID-19 hospitalized individuals showed that the EUR and SAS populations are characterized by a greater susceptibility to hospitalization than the EAS and AFR population. In fact, while 5.7% of EUR and 6.9% of SAS subjects exceed the OR threshold, a significantly lower percentage were observed in EAS (0.9%) and AFR (0%) populations (Figure 3) (Supplementary Table S1).

Altogether, these results confirm that AFR, EAS, EUR and SAS have different genetic risk for infection and respond differently to the SARS-CoV-2 infection and COVID-19 severity, as also demonstrated in previous studies [13,14,15,18]. Moreover, the presented data highlight the existence of a genetic basis for the observed variability among populations with respect to the SARS-CoV-2 infection and disease outcomes, which can range from COVID-19 positive without clinical manifestation to COVID-19 severe symptoms and hospitalization. The origin of this clinical spectrum remains unclear, although the genetic architecture of the host (including common and rare variants of *ACE2*, *IFNAR2*, *CXCR6* and *TLR7* genes) and non-genetic features (such as comorbidities, age, sex and lifestyle) have been shown to influence the COVID-19 outcomes [12,18]. To this purpose, the human genetics community launched the COVID-19 Host Genetics Initiative [19], in order to detect specific genetic variants associated with different COVID-19 symptoms. The study will allow identifying people at high or low risk among worldwide populations. In addition, our statistical approach outlined differences among African, East Asian, European and South Asian populations in terms of genetic distribution of variants associated with COVID-19 infection and related outcomes. Our results show that African population may have a lower genetic susceptibility to SARS-CoV-2 infection and, consequently, being less prone to develop severe symptoms and no need for hospitalization. Instead, EAS, EUR and SAS populations display similar percentages of subjects at higher risk of infection and subjects who do not need hospitalization. Altogether, our results differ from data reported by Centers of Disease Control and Prevention (CDC) that utilized age as a key parameter for assessing the risk of infection, hospitalization and death. This discrepancy could be due to the fact that our data were not adjusted for age, rather we only considered the genetic distribution of the SNPs set (rs73064425, rs2531743, rs143334143, rs879055593, rs2109069, rs2236757) among different populations. Furthermore, taking into account severe symptoms and COVID-19 hospitalization, this study outlines differences between EAS and EUR. In particular, the European population displays a higher percentage of subjects showing severe COVID-19 and hospitalization compared to East Asian population (Supplementary Table S1).

In conclusion, the characterization of the genetic distribution among populations and the study of COVID-19 clinical symptoms will offer new insights for understanding disease pathogenesis and clarify the different clinical responses. Moreover, the knowledge of the host’s genetic architecture will lead to the identification of biomarkers that can be used for the development of population-specific protocols and provide a more effective management of COVID19. Of course, the realization of such protocols should also include non-genetic factors known to contribute to COVID-19 infection and outcome, such as age, sex, exposure to the virus related to occupation, lifestyle, comorbidities and socioeconomic status. In this way, each country could benefit from a population-wide risk assessment in order to personalize their national vaccine programs and preventative measures, as well as from the allocation of resources and the access to proper therapeutic interventions. In particular, the knowledge of host genetic makeup could be crucial for the realization of personalized medicine protocols tailored to improve the management of patients suffering from COVID-19.

## Figures and Tables

**Table 1:** Association results of the comparison between the combined ORs and the different populations. AFR: African, EAS: East Asia, EUR: European, SAS: South Asian. NS: not significant.

Phenotype	Comparison.	*p*-Value	Adjusted *p*-Value
Infection risk	EUR vs. EAS	NS	NS
	EUR vs. AFR	1.116 × 10^−8^	2.678 × 10^−7^
	EUR vs. SAS	NS	NS
	EAS vs. AFR	2.764 × 10^−7^	5.529 × 10^−6^
	EAS vs. SAS	NS	NS
	AFR vs. SAS	4.912 × 10^−5^	7.368 × 10^−4^
COVID-19 hospitalized	EUR vs. EAS	4.724 × 10^−8^	1.039 × 10^−6^
	EUR vs. AFR	6.699 × 10^−11^	1.808 × 10^−9^
	EUR vs. SAS	2.645 × 10^−3^	2.381 × 10^−2^
	EAS vs. AFR	7.099 × 10^−4^	7.099 × 10^−3^
	EAS vs. SAS	3.331 × 10^−10^	8.326 × 10^−9^
	AFR vs. SAS	5.187 × 10^−12^	1.504 × 10^−10^
Severe illness	EUR vs. EAS	4.927 × 10^−4^	5.949 × 10^−3^
	EUR vs. AFR	4.609 × 10^−11^	1.290 × 10^−9^
	EUR vs. SAS	2.137 × 10^−5^	3.847 × 10^−4^
	EAS vs. AFR	2.408 × 10^−7^	5.058 × 10^−6^
	EAS vs. SAS	1.197 × 10^−10^	3.113 × 10^−9^
	AFR vs. SAS	1.036 × 10^−14^	3.110 × 10^−13^

## Data Availability

The data described in the present study are included within the manuscript and the Supplementary materials.

## Supplementary Materials

The following supporting information can be downloaded at: https://www.mdpi.com/article/10.3390/jpm12111851/s1, Table S1: Supplementary Table; Supplementary Methods.

## References

[1] World Health Organization (2022). COVID-19 Weekly Epidemiological Update.

[2] Liu Y.C., Kuo R.L., Shih S.R. (2020). COVID-19: The first documented coronavirus pandemic in history. Biomed. J..

[3] Caputo V., Termine A., Fabrizio C., Calvino G., Luzzi L., Fusco C., Ingrascì A., Peconi C., D’Alessio R., Mihali S. (2021). Age and Sex Modulate SARS-CoV-2 Viral Load Kinetics: A Longitudinal Analysis of 1735 Subjects. J. Pers. Med..

[4] Strafella C., Caputo V., Termine A., Barati S., Gambardella S., Borgiani P., Caltagirone C., Novelli G., Giardina E., Cascella R. (2020). Analysis of ACE2 Genetic Variability among Populations Highlights a Possible Link with COVID-19-Related Neurological Complications. Genes.

[5] Long S. (2021). SARS-CoV-2 Subgenomic RNAs: Characterization, Utility, and Perspectives. Viruses.

[6] Qin L., Meng J., Ding X., Jiang T. (2022). Mapping Genetic Events of SARS-CoV-2 Variants. Front. Microbiol..

[7] Caputo V., Calvino G., Strafella C., Termine A., Fabrizio C., Trastulli G., Ingrascì A., Peconi C., Bardini S., Rossini A. (2022). Tracking the Initial Diffusion of SARS-CoV-2 Omicron Variant in Italy by RT-PCR and Comparison with Alpha and Delta Variants Spreading. Diagnostics.

[8] Strafella C., Caputo V., Termine A., Barati S., Caltagirone C., Giardina E., Cascella R. (2020). Investigation of Genetic Variations of IL6 and IL6R as Potential Prognostic and Pharmacogenetics Biomarkers: Implications for COVID-19 and Neuroinflammatory Disorders. Life.

[9] Luo X., Lv M., Zhang X., Estill J., Yang B., Lei R., Ren M., Liu Y., Wang L., Liu X. (2022). Clinical manifestations of COVID-19: An overview of 102 systematic reviews with evidence mapping. J. Evid. Based Med..

[10] Cummings M.J., Baldwin M.R., Abrams D., Jacobson S.D., Meyer B.J., Balough E.M., Aaron J.G., Claassen J., Rabbani L.E., Hastie J. (2020). Epidemiology, clinical course, and outcomes of critically ill adults with COVID-19 in New York City: A prospective cohort study. Lancet.

[11] Shelton J.F., Shastri A.J., Ye C., Weldon C.H., Filshtein-Sonmez T., Coker D., Symons A., Esparza-Gordillo J., Aslibekyan S., 23andMe COVID-19 Team (2021). Trans-ancestry analysis reveals genetic and nongenetic associations with COVID-19 susceptibility and severity. Nat. Genet..

[12] Horowitz J.E., Kosmicki J.A., Damask A., Sharma D., Roberts G.H.L., Justice A.E., Banerjee N., Coignet M.V., Yadav A., Leader J.B. (2022). Genome-wide analysis provides genetic evidence that ACE2 influences COVID-19 risk and yields risk scores associated with severe disease. Nat. Genet..

[13] Clark A., Jit M., Warren-Gash C., Guthrie B., Wang H.H.X., Mercer S.W., Sanderson C., McKee M., Troeger C., Ong K.L. (2020). Centre for the Mathematical Modelling of Infectious Diseases COVID-19 working group. Global, regional, and national estimates of the population at increased risk of severe COVID-19 due to underlying health conditions in 2020: A modelling study. Lancet Glob. Health.

[14] Hashim M.J., Alsuwaidi A.R., Khan G. (2020). Population Risk Factors for COVID-19 Mortality in 93 Countries. J. Epidemiol. Glob. Health.

[15] Marçalo R., Neto S., Pinheiro M., Rodrigues A.J., Sousa N., Santos M.A.S., Simão P., Valente C., Andrade L., Marques A. (2022). Evaluation of the genetic risk for COVID-19 outcomes in COPD and differences among worldwide populations. PLoS ONE.

[16] Cunningham F., Amode M.R., Barrell D., Beal K., Billis K., Brent S., Carvalho-Silva D., Clapham P., Coates G., Fitzgerald S. (2015). Ensembl 2015. Nucleic. Acids Res..

[17] Hollander M., Wolfe D.A., Chicken E. (1999). Nonparametric Statistical Methods.

[18] Booth A., Reed A.B., Ponzo S., Yassaee A., Aral M., Plans D., Labrique A., Mohan D. (2021). Population risk factors for severe disease and mortality in COVID-19: A global systematic review and meta-analysis. PLoS ONE.

[19] COVID-19 Host Genetics Initiative (2020). The COVID-19 Host Genetics Initiative, a global initiative to elucidate the role of host genetic factors in susceptibility and severity of the SARS-CoV-2 virus pandemic. Eur. J. Hum. Genet..

