# Supplementary material for: Analysis of Genetic Variants Associated with COVID-19 Outcome Highlights Different Distributions among Populations
Source: J Pers Med. 2022 Nov 5;12(11):1851. doi: 10.3390/jpm12111851 (PMC9692526; doi:10.3390/jpm12111851)
Supplement: Supplementary file 1 [file jpm-12-01851-s001.zip › Supplementary methods.pdf]

Supplementary Methods

Here is a detailed explanation for the calculation of the combined OR:

In mathematics, an element (or member) of a set is any one of the distinct objects that belong to that set. In our case the SNPs set has 6 elements (rs143334143, rs2109069, rs2236757, rs2531743, rs73064425, rs879055593). Each subject has an OR for each of the SNPs. Capital pi notation (Π) indicates the product of a sequence of factors, where the subscript (in our case k) is the index of multiplication, namely the indicator of the element in the set to be multiplied. Therefore,

$$\prod_{k=1}^{n} OR_k = OR_1 \cdot OR_2 \cdot OR_3 \cdot OR_4 \cdot OR_5 \cdot OR_6$$

Let's consider an example subject for our calculation. This subject has the following OR for the SNPs set:

**EXAMPLE SUBJECT OR VALUES FOR THE SNPs SET**						
**SNP**	**rs143334143**	**rs2109069**	**rs2236757**	**rs2531743**	**rs73064425**	**rs879055593**
**OR**	0.7251	1.055	0.8583	1.0482	0.5885	0.8190

Applying the product of a sequence for these ORs gives the combined OR value:

$$\prod_{k=1}^{n} OR_k = 0.7251 \cdot 1.055 \cdot 0.8583 \cdot 1.0482 \cdot 0.5885 \cdot 0.8190 \; = 0.3318$$

This calculation has been done for each subject in our data.
